# The complete mitochondrial genome of *Semblis atrata* (Trichoptera: Phryganeidae)

**DOI:** 10.1080/23802359.2022.2080595

**Published:** 2022-06-09

**Authors:** Enzhi Liu, Junjian Li, Siyang Ou, Bingjun Dong, Baotian Yang, Yu Zhou

**Affiliations:** College of Life Sciences, Shenyang Normal University, Shenyang, China

**Keywords:** Illumina sequencing, mitogenomics, phylogeny, *Semblis atrata*

## Abstract

*Semblis atrata* is one of three *Semblis* species distributed in clean brooks and streams in northern Eurasia. Genomic DNA of an *S. atrata* sample was extracted and sequenced for assembly and annotation of its complete mitogenome. The complete mitochondrial genome of *S. atrata* was 14,909 bp in length and consisted of 13 protein-coding genes, 2 rRNA genes, and 22 tRNA genes. The *S. atrata COX1* gene features a CGA start codon, and *COX1*, *COX2, ND1*, and *ND5* exhibit incomplete stop codons that are presumed to be completed by the addition of 3’ A residues to the mRNA. The nucleotide composition was highly AT biased, accounting for 77.71% of the whole mitogenome. Phylogenetic analysis placed *Semblis* as sister to *Eubasilissa*. The complete mitochondrial genome will be helpful for further studies on the population genetics of this species and phylogenetic analyses of Trichoptera.

The genus *Semblis* within family Phryganeidae has the closest phylogenetic relationship with *Eubasilissa* according to molecular phylogenetic evidence (Thomas et al. 2020)*. Semblis atrata* Gmelin 1789 is an obscure species distributed throughout northern Eurasia. The larvae of *S. atrata* predominantly inhabit slow-flowing brooks and streams with clear, humic, oxygen-rich, slightly acidic water and stony sand bottoms. Adults of *S. atrata* ubiquitously fly around such brooks and streams (Berglind et al. 1999). Currently, the only published mitochondrial DNA sequences of *Semblis* species are partial *COX1* gene sequences, which have been used for DNA barcoding or multi-gene phylogenetic research (Zhou et al. 2016; Thomas et al. 2020). The complete mitochondrial genome of *S. atrata* is thus of great importance for shedding light on the diversity and phylogenetic relationships of Trichoptera.

The research materials were collected from Hushan village, Anshan, Liaoning Province, China (123.460°E, 40.787°N) in May 2021. After collection, specimens were immediately preserved in 95% ethanol until DNA extraction. A voucher specimen was deposited at Shenyang Normal University, Shenyang, China (Yu Zhou is the contact person: zhouyu1988@outlook.com) under voucher number ZY-2021050501. Total genomic DNA was isolated using the TIANamp Genomic DNA Kit (TIANGEN Biotech) according to the manufacturer’s instructions. The sequencing library was prepared following McCullagh and Marcus (2015) and sequenced by the Illumina HiSeq 2500 platform with 150 bp paired-end sequencing (Sangon Biotech, Shanghai, China). The mitochondrial genome was assembled *de novo* using NOVOPlasty (Dierckxsens et al. 2017) with a partial mitochondrial *COX1* sequence of *S. atrata* (GenBank: KX107215) (Zhou et al. 2016) used as the seed sequence. The mitochondrial genome was annotated using the MITOS Webserver (Bernt et al. 2013) with reference to the complete *Eubasilissa regina* mitogenome (GenBank: NC023374). The annotated mitogenome was submitted to GenBank under accession number MZ514855. For each of the 13 protein-coding genes (PCGs), DNA sequences were aligned using PRANK (Löytynoja 2014) with the ‘-mttranslate’ alignment option. The alignments were further refined using Gblocks 9.1 b (Castresana 2000) with the ‘codon’ model and other default settings. All refined alignments were then concatenated into the final data set. For the concatenated data set, we manually defined three partitioning strategies: unpartitioned, three partitions (one partition for each codon position), and 13 partitions (one partition for each PCG). Comparisons of the three partitioning strategies and selection of corresponding nucleotide substitution models were conducted with the Bayesian information criterion implemented in PartitionFinder (Lanfear et al. 2012). The 3-partition scheme (one partition for each codon position) was chosen as the best-fitting partitioning strategy, and all three partitions favored the GTR + Γ + I model. The phylogenetic relationships between *Semblis* and 19 other Trichoptera species were reconstructed using maximum likelihood (ML) analysis. Two species from the sister order Lepidoptera were included as outgroups (Figure 1). The ML tree was estimated by using RAxML version 8.0 (Stamatakis 2014) with the ‘GTRGAMMAI’ model. Support for nodes in the ML tree was assessed with a rapid bootstrap analysis (option -f a) with 1,000 replicates.

**Figure 1.**
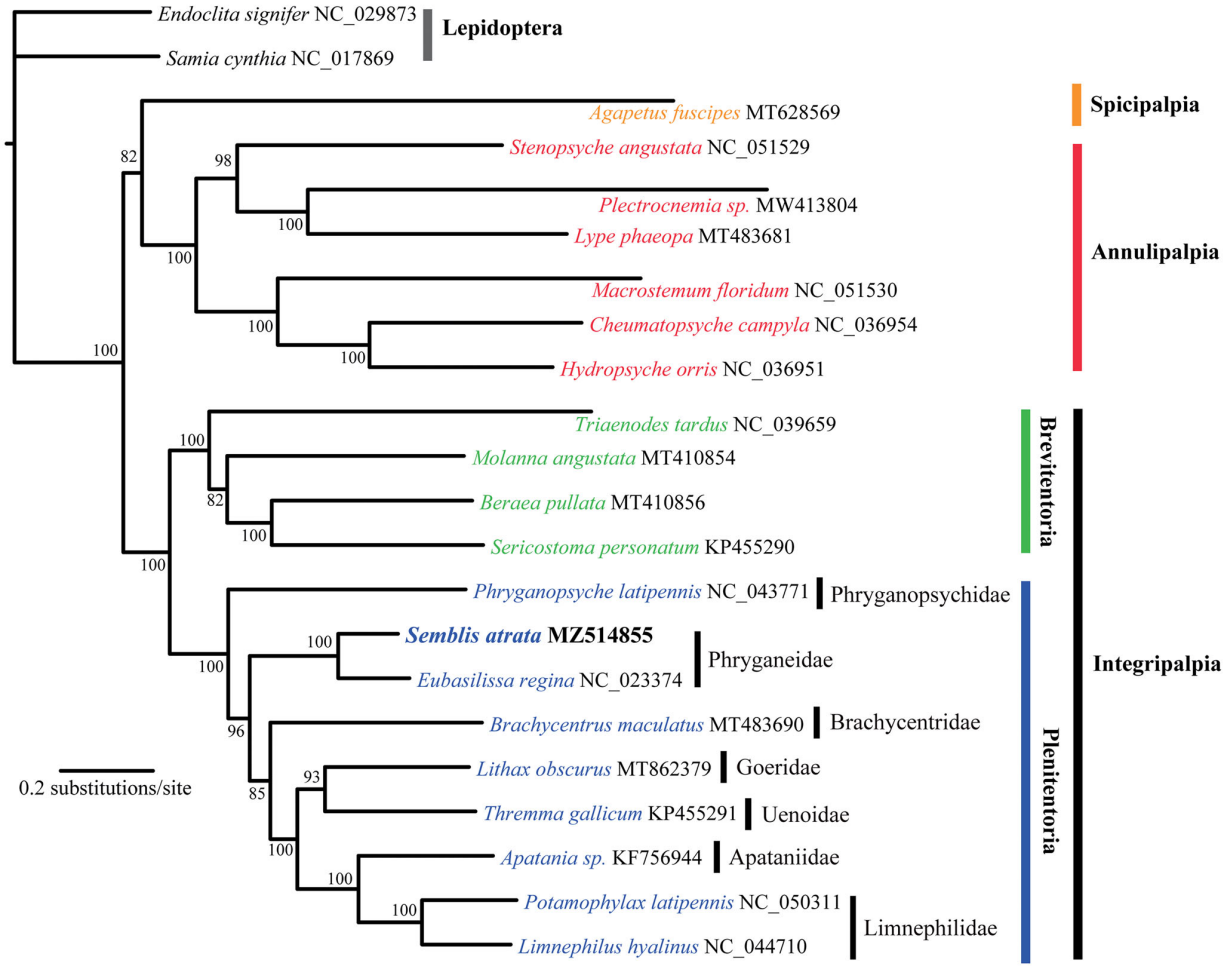
The maximum likelihood (ML)-based phylogenetic relationships of twenty Trichoptera species based on 13 protein-coding genes.

The circular mitochondrial genome of *S. atrata* is 14,909 bp in length. According to the annotation results, it contains 13 PCGs, 22 tRNA genes, and 2 rRNA genes, which is typical of animal mitogenomes (Boore 1999). The nucleotide composition of this mitogenome is A 39.11%, C 14.11%, G 8.18%, and T 38.60%, with a higher A + T content (77.71%) than G + C content (22.29%). These characteristics of the mitogenome are similar to those of other Phryganeidae mitogenomes (Wang et al. 2013; Zhou et al. 2016). The mitochondrial gene composition and order in *S. atrata* are identical to those in most other Trichoptera mitogenomes (Wang et al. 2013; Living Prairie Mitogenomics Consortium 2018; Marcus 2018). Four of the protein-coding genes (*COX1*, *COX2*, *ND1*, and *ND5*) have single-nucleotide stop codons (T) completed by posttranscriptional addition of 3′ A residues. Similar to that in many other insects, *COX1* in *S. atrata* begins with a start codon (CGA) (Liao et al. 2010). The gene arrangement and length of tRNA and rRNA regions are typical for Trichoptera (Wang et al. 2013; Lalonde and Marcus 2017; Living Prairie Mitogenomics Consortium 2018).

Phylogenetic analysis revealed the monophyly of Trichoptera, and all suborders and infraorders were monophyletic (Figure 1). The phylogenetic tree showed that *Semblis* is a sister group to *Eubasilissa* with maximal nodal support. The results thus validated the close relationship between the two genera, and the same results have been reported by Thomas et al. (2020). The complete mitogenome of *S. atrata* presented here will be useful for investigating the phylogeny and population genetics of Phryganeidae and even Trichoptera.

## Ethics statement

The study protocol was approved by the Committee on the Ethics of Animal Experiments of the College of Life Sciences, Shenyang Normal University.

## Author contributions

Y. Z. designed and conceived this work; E. L., S. O., B. D. and B. Y. collected the samples; E. L. and J. L. wrote the first version of the manuscript; Y. Z., E. L. and J. L. carried out the investigation. All authors read, revised and approved the final manuscript.

## Data Availability

The genome sequence data that support the findings of this study are openly available in GenBank of the NCBI at https://www.ncbi.nlm.nih.gov/ under accession no. MZ514855. The associated BioProject, SRA, and Bio-Sample numbers are PRJNA752916, SRR15371432, and SAMN20667750, respectively.
